# Ranchers’ Preferences for Grazing Programs in the Voluntary Carbon Market: Insights from a Discrete Choice Experiment in the Great Plains and Front Range, USA

**DOI:** 10.1007/s00267-026-02450-z

**Published:** 2026-04-11

**Authors:** Nicole Nimlos, Christopher Bastian, John Derek Scasta

**Affiliations:** 1 Ecosystem Science and Management, University of Wyoming, Laramie, WY USA; 2 Agricultural and Applied Economics, University of Wyoming, Laramie, WY USA; 3 Laramie Research and Extension Center, Agricultural Experiment Station, Laramie, WY USA

**Keywords:** Carbon credit, Carbon offset, Grasslands, Multinomial regression, Rangelands, Soil carbon

## Abstract

Emerging opportunities in the voluntary carbon market raise important questions about ranchers’ preferences for carbon programs requiring altered grazing practices. Accordingly, we conducted the first study to evaluate ranchers’ preferences for the design of three contemporary carbon programs administered by the American Carbon Registry, Climate Action Reserve, and Verra. We surveyed 506 ranchers across ten states in the Front Range and Great Plains, USA, encompassing more than 1.7 million acres. Ranchers exhibited differing levels of interest across the carbon programs, with the majority opting out of enrollment. Among those willing to participate, ranchers preferred programs with shorter contract lengths, an established track record of selling carbon credits, reliance on soil sampling (rather than conservation easements) to estimate soil carbon change, and higher payment amounts. Although there was no preference between annual versus lump sum payments, greater payment levels significantly increased the likelihood of enrollment. On average, respondents who chose to participate in a carbon program required $12.17 per acre with contract renegotiation every 2.87 years at the time of the survey. To increase participation, carbon companies should clearly communicate management requirements and financial benefits, highlight non-financial benefits, and build trust through greater transparency.

## Introduction

The voluntary carbon market (hereafter ‘carbon market’) offers a new opportunity for ranchers to potentially generate additional income while continuing their livestock operations and improving their land management practices. To participate, ranchers partner with a project developer (or carbon company) to adopt land management practices that promote increased soil carbon sequestration – the process of removing atmospheric carbon dioxide (CO_2_) through photosynthesis and storing it in the soil (Ontl and Schulte 2012). These practices must be additional to current management and may include increasing grazing rotations and pasture rest, enrolling in conservation easements when in at-risk areas to conversion, or applying organic fertilizers.

Project developers work directly with registries, who are the entities that create the methodologies for generating and verifying carbon credits (Nimlos et al. 2025b). One carbon credit is equal to one metric ton of CO_2_ equivalents sequestered into the soil or prevented from being released (e.g., through tilling, urban development, etc.). Project developers oversee the verification and certification process by a third-party, which determines the number of carbon credits to be issued based on soil sampling or modeling. Once issued, the project developer sells the carbon credits to buyers who are seeking to offset their emissions (for example, companies in the travel and energy sectors). Ranchers receive a portion of the revenue from the carbon credit sales with the project developer to offset the initial project investment. The payment amount and frequency can vary and are specified in a contract between the rancher and the project developer.

American Carbon Registry (hereafter ‘ACR’), Climate Action Reserve (hereafter ‘CAR), and Verra are the three prominent registries that have developed carbon program methodologies tailored to grassland management. Although there are numerous protocols relating to grassland and livestock management in the market, we explore three protocols under these registries, including ACR’s *Avoided Conversion of Grasslands and Shrublands to Crop Production*, CAR’s *U.S. Grassland Protocol*, and Verra’s *VM0032 Methodology for the Adoption of Sustainable Grasslands through Adjustment of Fire and Grazing* methodology. These methodologies under ACR and CAR require ranchers to avoid the conversion of grasslands to croplands through long-term conservation agreements, typically by enrolling land in a conservation easement and restricting its use to grazing. These programs offer 40 and 100 year contracts, respectively, and rely on modeling rather than soil sampling to estimate greenhouse gas emission reductions from avoided land conversion (Climate Action Reserve 2020). There is currently one project enrolled under ACR’s methodology, covering more than 27,000 acres with a crediting time period that extends through 2049 as of March 9, 2026. There are currently 26 projects registered under CAR’s methodology.

Verra’s *VM0032 Methodology for the Adoption of Sustainable Grasslands through Adjustment of Fire and Grazing* requires ranchers to increase grazing rotations and pasture rest. These contracts range from 20 to 40 years and do not require enrollment in a conservation easement. Instead, they rely on soil sampling to track changes in soil organic carbon resulting from improved grazing management practices. These changes must be reassessed every five years to determine the number of carbon credits to be issued. There are currently 17 projects under development, but no projects registered under this methodology in the United States (U.S.) as of March 9, 2026.

Low payment levels, strict program requirements, and long contract lengths are common barriers to producer participation in carbon markets with variability noted across location and land uses (Thompson et al. 2022; Barbato and Strong 2023; Nimlos et al. 2025a). In the United Kingdom, farmers preferred receiving carbon payments over time rather than as a lump sum upfront (Phelan et al. 2024). U.S. row crop producers reported high levels of uncertainty surrounding carbon markets, related polices, and economic outcomes (Han and Niles 2023). Research in forest carbon markets suggests management objectives, contract length, property size, management plan requirements, and land restrictions all influence a landowner’s willingness to participate (Sharma and Kreye 2022). More broadly, landowners tend to be cautious about enrolling in conservation easements due to concerns about permanence and land-use restrictions (Farmer et al. 2016; Miller et al. 2010).

Research on ranchers’ preferences for grazing related carbon programs remains limited. In earlier research, we found that 55% of ranchers expressed interest in participating in grazing-related carbon markets (Nimlos et al. 2025a). Ranchers were more likely to participate if they owned more private acreage, produced hay, were enrolled in a conservation easement, participated in a conservation program in the past, believed their community would support their participation, or reported limited knowledge about the market (Nimlos et al. 2025a; Nimlos et al. manuscript under review). In contrast, older producers involved in crop production and those skeptical of the carbon market were less likely to participate. This study builds on our previous work by examining ranchers’ preferences for the design of three specific contemporary carbon programs in a discrete choice experiment, rather than only their willingness to participate in the carbon market. We also explore preferred payment amounts and schedules (i.e., annual vs. lump sum payments), as well as whether certain rancher characteristics influence program choice.

## Methods

### Study Area

In January of 2024, we administered a survey to ranchers across ten states in the U.S. Great Plains and Front Range region, including Colorado, Kansas, Montana, Nebraska, New Mexico, North Dakota, Oklahoma, Texas, South Dakota, and Wyoming. Livestock grazing is a dominant land use in this region and is increasingly threatened by the conversion of native rangeland to cropland (Smart et al. 2021). We partnered with DTN (https://www.dtn.com/), a company that maintains data on over 95% of U.S. farmers and ranchers, to acquire a random sample of participants. Our inclusion criteria included individuals who own or manage 200 or more acres of rangeland, grassland, or shrubland, as they are more likely to meet eligibility requirements for carbon program enrollment. Some ranchers also engaged in crop, hay, or multiple types of livestock production. DTN provided email and mailing addresses for all potential participants.

**Fig. 1:**
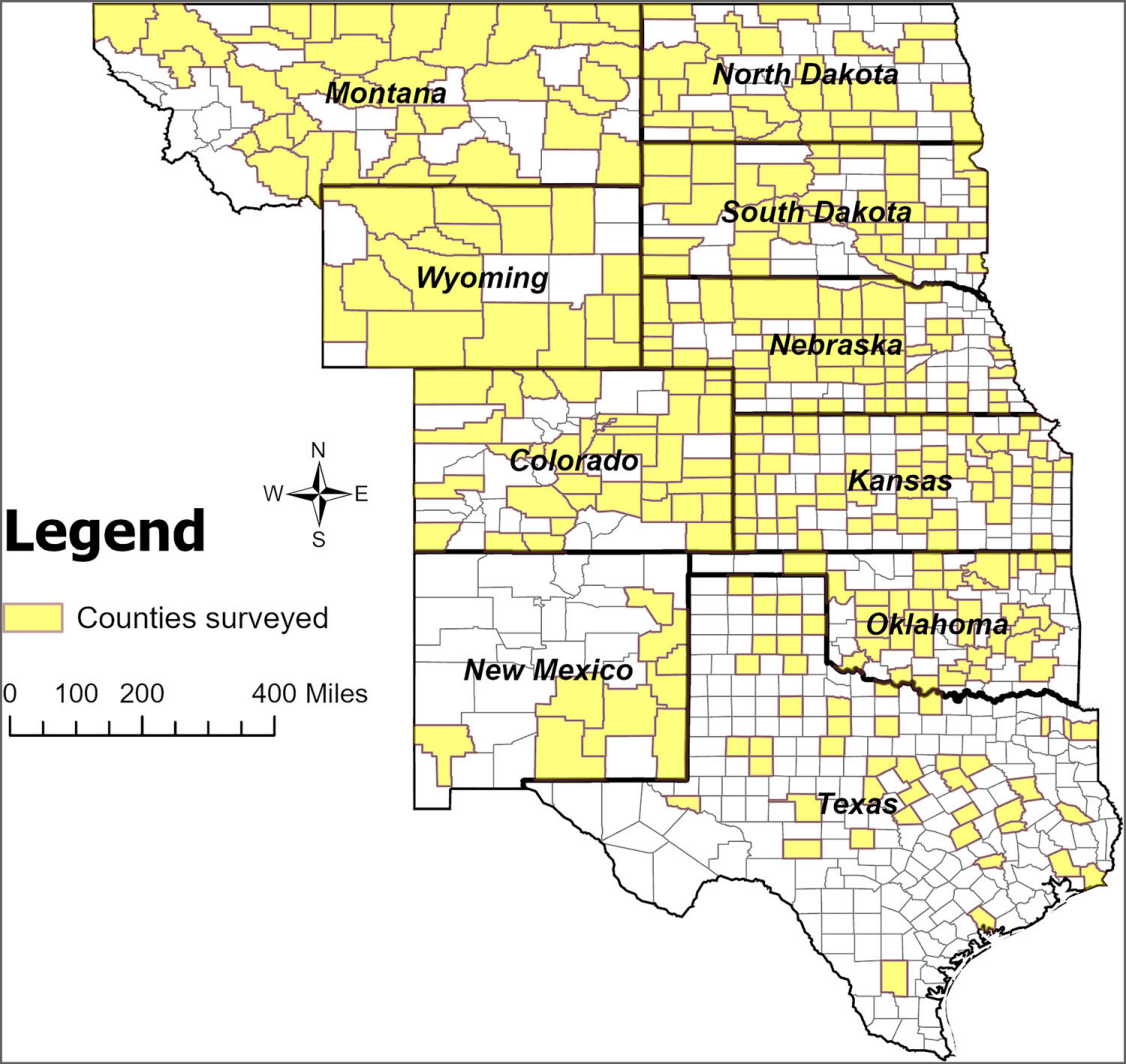
The ten states and counties surveyed in the Front Range and Great Plains, USA

The survey was equally distributed to 350 randomly selected ranchers in each state to obtain a representative sample from the population of ranchers in each state, resulting in a total of 3500 surveys administered. By the fall of 2024, we received 506 completed surveys and deemed 100 surveys invalid due to address changes or deceased respondents, resulting in a 15% response rate. Collectively, our survey encompasses over 1.7 million acres of publicly and privately managed grazinglands (Fig. 1).

### Questionnaire

The survey included discrete choice questions, which can be used to estimate how people value environmental goods or services with multiple attributes (or characteristics), such as conservation programs (Champ et al. 2017). We used choice experiments in which ranchers evaluated trade-offs among three carbon programs (ACR, CAR, and Verra) based on their respective attributes, including contract length, conservation easement requirements, soil testing requirements, and an established track record of selling carbon credits (Table 1).

**Table 1:** Attributes of the three contemporary carbon programs presented to respondents in the choice experiments

	Climate Action Reserve’s *U.S. Grassland Protocol* (CAR)	American Carbon Registry’s *Avoided Conversion of Grasslands and Shrublands to Crop Production* (ACR)	Verra’s *VM0032 Methodology for the Adoption of Sustainable Grasslands through Adjustment of Fire and Grazing* (Verra)
**Carbon program attributes:**			
Contract length	100 years	40 years	20–40 years
Conservation easement required	Yes	Yes	No
Soil must be tested every 5 years to demonstrate accumulation of carbon to earn carbon credits	No	No	Yes
Established track record of selling carbon credits	Yes	Yes	No

We prepared participants for the choice experiments by walking them through the steps involved in joining a carbon program and included “knowledge check” questions to maintain engagement. We informed them that they would have to establish a management plan that outlines the specific management changes they would implement to support carbon storage on their property, such as rotational grazing or halting tilling. We then presented respondents with the following preamble:

“Imagine you have been contacted by a carbon project developer. They have presented you with an opportunity to participate in a grassland carbon project and have offered you a contract for three different programs that you could enroll in. Additionally, the project developer has successfully secured a buyer who is interested in purchasing the carbon credits generated by your participation in the program. This means that your management efforts will have a tangible value in the market. Below, you will find the characteristics of three different grassland carbon programs. You negotiate the payment frequency and amount with the project developer based on your preferences and market demand. The next 4 questions are hypothetical scenarios. Consider each one separately.”

The carbon programs presented in the survey reflected the actual enrollment requirements of ACR’s *Avoided Conversion of Grasslands and Shrublands to Crop Production*, CAR’s *U.S. Grassland Protocol*, and Verra’s *VM0032 Methodology for the Adoption of Sustainable Grasslands through Adjustment of Fire and Grazing* methodologies. We held all carbon program attributes constant according to their respective registries and varied only the payment amount and frequency in the choice experiments considering these vary by contract. We included four options for payment frequency in the choice experiments, including annual payments negotiated every three years or five years and lump sum payments negotiated every three years or five years. For annual payments, we assigned four possible amounts: $1, $2, $4, or $6 per acre enrolled. These amounts were lower because they reflected payments distributed annually over the duration of the contract. For lump sum payments tied to a three-year contract, the options were $3, $6, $12, or $18 per acre. Because five-year contracts span a longer duration, the lump sum payment options were higher: $5, $10, $20, or $30 per acre. Payment frequencies were developed in consultation with carbon market experts, and payment amounts were based on carbon credit prices reported by Ecosystem Marketplace (2022), a widely recognized source for carbon market data.

We used SAS statistical software to generate full factorial optimal designs for payment amount and payment frequency. Full factorial designs ensure that main and interaction effects are statistically independent (orthogonal), allowing unbiased estimation of their influence (Champ et al. 2017). Orthogonal coding assigns values to attribute levels so that the sum of values in each column (representing main or interaction effects) equals zero (Hensher et al. 2005). We selected the optimal choice experiment designs with the highest D-efficiency, which assumes all carbon programs are equally attractive and preference parameters are equal to zero (Champ et al. 2017). This design resulted in 16 unique discrete choice questions. Each respondent only received four discrete choice questions to reduce respondent fatigue and improve the stability of preference weighting (DeShazo and Fermo 2002; Day et al. 2012). Respondents could choose to opt-out of any or all of the choice questions, resulting in four observations per respondent. Four survey versions were distributed equally across participants in each state, with each version containing four different choice experiments. Respondents were randomly assigned to one of the four versions. In each choice experiment, respondents evaluated the three carbon programs, with all program attributes held constant and only the payment amount and payment frequency varying.

**Fig. 2:**
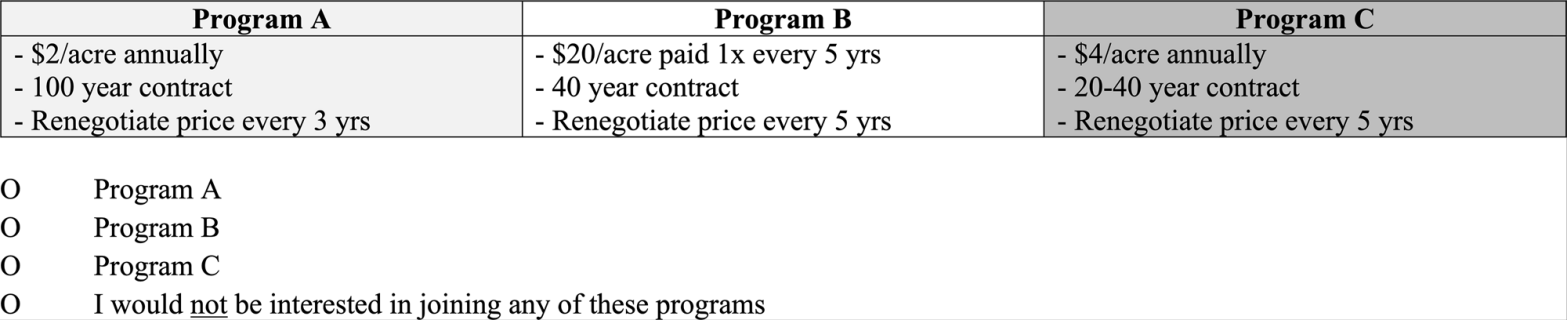
An example choice question presented in the survey

After reviewing each carbon program’s requirements (Table 1), we asked respondents to indicate their willingness to enroll in one of the three carbon programs based on the payment frequency and amount, or to opt-out by selecting “I would not be interested in joining any of these programs” (Fig. 2). Following Random Utility Theory, it is expected that each respondent will choose the option that maximizes their utility (satisfaction) on a given choice occasion (Louviere, Hensher and Swait 2000). Following the choice experiments, we asked respondents Likert scale questions regarding how carbon program attributes affected their willingness to participate (strongly disagree, disagree, neutral, agree, strongly agree). Lastly, we asked respondents to indicate their age, property size, whether they are enrolled in a conservation easement, have previously participated in a conservation program, and if they engage in hay or crop production.

### Survey Implementation

Following a modified Dillman approach (Dillman 2000), we first emailed each respondent to invite them to participate in the study and notify them that a paper survey would be mailed. Each participant then received the paper survey with a prepaid return envelope, along with the option to complete it online via *Qualtrics*. After the initial mailing, we sent three follow-up email reminders at two-week intervals to encourage responses. This study was reviewed and determined to be exempt by the University of Wyoming’s Institutional Review Board on September 23rd, 2023.

## Data Analysis

The choice experiment questions allowed us to assess ranchers’ preferences for each carbon program’s attributes, including contract length, requirement to enroll in a conservation easement, requirement to demonstrate soil carbon accumulation, presence of an established track record of selling carbon credits, payment amount, and payment frequency. We coded these attributes based on the carbon program selected by the respondent (Table 2). For example, if a respondent selected CAR (Program A), the variables were coded: contract = 100 years, easement=yes, accumulation = no, and record=yes. For ACR (Program B): contract = 40 years, easement=yes, accumulation=no, and record=yes. For Verra (Program C): contract = 20–40 years, easement = no, accumulation = yes, and record = no. We coded payment amount and payment frequency according to the values presented in each choice experiment. Each respondent contributed four rows of data corresponding to the four choice experiments they completed. The dependent variable was the program they selected (1 = CAR, 2 = ACR, 3 = Verra, 4 = opt-out) and we set the opt-out as the reference level. We conducted a chi-square test to determine whether we received proportionally the same number of responses from each of the four survey versions (α > 0.05 indicating equal proportions). We conducted all data analysis in R software.

**Table 2:** The coding scheme for the carbon program attributes in the choice experiments

	CAR	ACR	Verra	Opt-out
**Variable**				
**Selection** (the respondent’s selection in the choice experiment)	1	2	3	4
**Contract** (contract length)	100 years	40 years	20–40 years	0
**Easement** (whether the carbon program requires ranchers to enroll in a conservation easement or not)	Yes	Yes	No	No
**Accumulation** (whether the carbon program requires demonstration of soil carbon accumulation to be issued carbon credits or not)	No	No	Yes	No
**Record** (whether the carbon program has an established track record of selling carbon credits or not)	Yes	Yes	No	No
**Pay_amount** (the payment amount for the carbon program)	Varied between each choice experiment, but ranged from $1/acre to $30/acre			
**Pay_frequency** (the frequency of the payment for the carbon program)	Varied between each choice experiment, but could be annually, every 3 years, or every 5 years			

### Random Utility Theory

We use the random utility model (RUM) to analyze the choice questions. RUM assumes that individual *n* faces a choice among *J* alternatives in one or more repeated choice experiments (Mariel et al. 2020). RUM assumes that individuals behave in a way that maximizes their utility, selecting the option in the choice experiment that provides the greatest satisfaction. The individual obtains an indirect utility ${U}_{{njt}}$ from alternative *j* in choice experiment *t*. Alternative *i* is chosen by individual *n* in choice experiment *t* if and only if ${U}_{{nit}} > {U}_{{njt}}$ for all $j{\ne }i$. The utility ${U}_{{njt}}$ can be decomposed into a deterministic (or representative) component ${V}_{{njt}}$ and a random error term ${\varepsilon }_{{njt}}$ (Eq. 1).

1 $${U}_{{njt}}={V}_{{njt}}+{\varepsilon }_{{njt}}$$

The deterministic component of the utility can be expressed as:

2 $${V}_{j}={\beta }^{{\prime} }{X}_{j}$$

where ${X}_{j}$ is a vector of variables associated with alternative *j* and $\beta$ is a vector of coefficients (Bastian et al. 2017). In the context of our study, when respondents are presented with three carbon program options and an opt-out (*j* = 4), their choice behavior can be modeled using RUM, where the systematic utility for each alternative depends on the program attributes (e.g., contract length, payment amount and payment frequency; Bastian et al. 2017; McFadden 1976). Our analysis differs from traditional discrete choice models, as we focused on producers’ preferences for existing carbon programs rather than hypothetical ones. We held attributes constant for each respective carbon program, while payment amount and payment frequency were varied to reflect differences among project developers. This approach provides insight into producers’ preferences for current carbon programs. We also included whether any part of their property was enrolled in a conservation easement (1 = yes, 0 = no) in the model selection process to assess whether existing easements influence these preferences.

Before modeling, we assessed for potential issues of multicollinearity among the candidate independent variables (Table 3), considering variables with a correlation coefficient greater than 0.6 to be highly correlated (Jorns et al. 2022). We found a high correlation between respondents’ level of agreement that payment frequency and payment amount impacted their willingness to enroll in a carbon program. Accordingly, we estimated three random utility models to examine how carbon program attributes and potential enrollment in a conservation easement (model 1), payment amount (model 2), and payment frequency (model 3) predict enrollment in the carbon market using the *multinom()* function from the *nnet* package in R (Venables and Ripley 2002). Multinomial regression is appropriate when the outcome variable is nominal with more than two unordered categories (Academic Success Center 2025).

**Table 3:** The candidate independent variables assessed for inclusion in the first multinomial regression model exploring respondents’ willingness to join the carbon market versus opting out

Model candidate variable	Description
Affect_easement	Level of agreement that the requirement to enroll in a conservation easement impacted their willingness to enroll in a carbon program
Affect_contract	Level of agreement that the contract length impacted their willingness to enroll in a carbon program
Affect_accumulate	Level of agreement that the requirement to demonstrate soil carbon accumulation impacted their willingness to enroll in a carbon program
Affect_record	Level of agreement that an established track record of selling carbon credits impacted their willingness to enroll in a carbon program
Affect_amount	Level of agreement that the payment amount impacted their willingness to enroll in a carbon
Affect_frequency	Level of agreement that the payment frequency impacted their willingness to enroll in a carbon program
Easement	Whether any part of their land was enrolled in a conservation easement or not

Note: The first six variables listed in the table are from the survey question “To what extent do you agree that the following items would affect your willingness to enroll into a carbon market program?” and were coded as -2=strongly disagree, -1=disagree, 0=neutral, 1=agree, 2=strongly agree. The last variable came from the survey question “Is any part of your land under a conservation easement?” and was coded as 1=yes, 0=no

We set the opt-out as the reference category for simpler interpretation, and so the odds of selecting each program are compared to the odds of opting out. The multinomial model can be generalized as (Bilder and Loughin 2024):

3 $$\log \left(\frac{{\pi }_{j}}{{\pi }_{{opt}-{out}}}\right)={\beta }_{j0}+{\beta }_{j1}{X}_{1}+\ldots +{\beta }_{{jp}}{X}_{p}$$

where ${\pi }_{j}$ is the probability of selecting carbon program *j* relative to ${\pi }_{{opt}-{out}}$, which is the probability of opting out, and ${X}_{1}$,…, ${X}_{p}$ are the predictor variables. The first model included the non-correlated explanatory variables detailed in Table 3 and took the form:

4 $$\begin{array}{l}\log \left(\frac{{\pi }_{j}}{{\pi }_{{opt}-{out}}}\right)={\beta }_{j0}+{\beta }_{j1}{affec}{t}_{{easement}}+{\beta }_{j2}{affec}{t}_{{contract}}\\\qquad\qquad\quad\,\,\,+{\beta }_{j3}{affec}{t}_{{accumulate}}+{\beta }_{j4}{affec}{t}_{{record}}+{\beta }_{j5}{Easement}\end{array}$$

The second model had payment frequency as the only explanatory variable and took the form:

5 $$\log \left(\frac{{\pi }_{j}}{{\pi }_{{opt}-{out}}}\right)={\beta }_{j0}+{\beta }_{j1}{pay}\_{frequency}$$

The third model had payment amount as the only explanatory variable and took the form:

6 $$\log \left(\frac{{\pi }_{j}}{{\pi }_{{opt}-{out}}}\right)={\beta }_{j0}+{\beta }_{j1}{pay}\_{amount}$$

The probability ${\Pr }_{j}$ that a respondent selected any of the three programs is found by exponentiating the log odds from the multinomial regression equation, such as:

7 $${\Pr }_{j}=\frac{\exp ({\beta }_{j0+}{\beta }_{j1}{X}_{1}+\ldots +{\beta }_{{jp}}{X}_{p})}{{\Sigma }_{k=1}^{J}\exp ({\beta }_{k0+}{\beta }_{k1}{X}_{1}+\ldots +{\beta }_{{kp}}{X}_{p})}$$

### Odds Ratios

We calculated Wald 95% confidence intervals for the regression coefficients and derived odds ratios by exponentiating these coefficients. Odds ratios help interpret the likelihood of a respondent selecting a carbon program given a one-unit increase in an independent variable (e.g., payment) relative to opting out. The odds ratio for selecting program *j* compared to opting out (or any reference category) can be found by exponentiating the estimated regression coefficients, such as:

8 $${{\rm{OR}}}_{j}=\exp ({\beta }_{j0}+{\beta }_{j1}{x}_{1}+\ldots +{\beta }_{{jp}}{x}_{p})$$

where an odds ratio ${{\rm{OR}}}_{j}$ less than one indicates reduced odds of selecting a carbon program compared to opting out, while an odds ratio greater than one suggests increased odds of selecting a carbon program (Tenny and Hoffman 2023). To quantify the change in odds of a rancher selecting each carbon program versus opting out, we used the following equations:

9 $${Percent}\,{decrease}=\left(1-{odds}\,{ratio}\right)x\,100 \%$$

10 $${Percent}\,{increase}=\left({odds}\,{ratio}-1\right)x\,100 \%$$

### Latent Class Analysis

We extended the multinomial regression analysis by estimating latent class models to account for unobserved heterogeneity in preferences and understand whether certain types of ranchers were more likely to prefer specific carbon programs (Mariel et al. 2020). We used predictor variables previously found to significantly influence willingness to join a carbon program to identify potential subgroups within the population that share certain characteristics, including age, enrollment in a conservation easement, participation in hay or crop production, past participation in a conservation program, and property size (Nimlos et al. 2025a). We coded property size as 1 for respondents owning less than the median (1000 acres) and 2 for those owning more. We coded age as 1 for respondents under 55 years old and 2 for those 55 or older. Enrollment in a conservation easement, participation in hay or crop production, and participation in a conservation program were coded as binary variables (1=yes, 0=no). Latent class models assume that preference parameters vary across a finite number of distinct classes, where each class represents a group of participants with similar choice behavior (Train 2009). The parameters are fixed within each class, but they differ between classes. Thus, the model estimates both the class-specific preference parameters and the probability that each respondent belongs to a given class.

Using the *poLCA* package in R (Linzer and Lewis 2011), we estimated the full latent class regression model and sequentially removed variables that did not show clear differentiation across classes. We assessed model fit by comparing Akaike’s Information Criterion (AIC), Bayesian Information Criterion (BIC), and chi-square values between the full and reduced models. Latent class analysis identifies subgroups within the data using a probabilistic framework that models the distribution of observed responses (Strzelecka et al. 2024). To determine the optimal number of classes, we compared AIC and BIC values across models ranging from two to six classes. The final model was selected based on the lowest AIC and highest chi-square value, indicating the best overall fit. The software then assigned each respondent to the class for which they had the highest probability of membership based on the final model.

Next, we estimated a multinomial regression to test whether latent class membership predicted carbon program selection and calculated the odds of respondents selecting one carbon program over another. We included the non-correlated explanatory variables from Table 3 and an interaction between payment amount and payment frequency. Because this model included only program selection (excluding the opt-out), we set the CAR program as the reference level so that the odds of selecting ACR or Verra are compared to the odds of selecting CAR. Thus, the fourth equation Eq. (11) models the probability of a respondent selecting ACR compared to CAR given the predictors and took the form:

11 $$\log \left(\frac{{\pi }_{{ACR}}}{{\pi }_{{CAR}}}\right)={\beta }_{j0}+{\beta }_{j1}{clas}{s}_{{membership}}+{\beta }_{j2}{affec}{t}_{{easement}}+{\beta }_{j3}{affec}{t}_{{contract}}+{\beta }_{j4}{affec}{t}_{{accumulate}}+{\beta }_{j5}{affec}{t}_{{record}}+{\beta }_{j6}{Easement}+{\beta }_{j7}{pay}\_{amount}* {pay}\_{frequency}$$

We also modeled the probability of a respondent selecting Verra compared to CAR given the predictors:

12 $$\begin{array}{l}{\mathrm{log}}\left(\frac{{\pi }_{{Verra}}}{{\pi }_{{CAR}}}\right)={\beta }_{j0}+{\beta }_{j1}{class}\_{membership}\\\qquad\qquad\quad\quad\,+\,{\beta }_{j2}{affect}\_{easement}+{\beta }_{j3}{affect}\_{contract}\\\qquad\qquad\quad\quad\,+\,{\beta }_{j4}{affect}\_{accumulate}+{\beta }_{j5}{affect}\_{record}\\\qquad\qquad\quad\quad\,+\,{\beta }_{j6}{Easement}+{\beta }_{j7}{pay}\_{amount}* {pay}\_{frequency}\end{array}$$

Finally, we examined whether respondents’ level of agreement that the various carbon program attributes impacted their willingness to join the carbon market differed by class membership.

## Results

The majority of respondents agreed or strongly agreed that the number of years required for enrollment (*n* = 309; 71%), payment amount (*n* = 277; 65%), payment frequency (*n* = 235; 55%), and the requirement to enroll in a conservation easement (*n* = 224; 51%) affected their willingness to enroll in a carbon program (Table 4). Respondents were neutral in their agreement that a carbon program’s established track record of selling carbon credits (*n* = 195; 46%) or the requirement to demonstrate soil carbon accumulation to earn carbon credits (*n* = 193; 45%) impacted their willingness to participate. Only 19% (*n* = 94) of respondents had any part of their land under a conservation easement (Table 4). We confirmed that the four survey versions were equally distributed among respondents (χ^2^ = 0.32; *p* = 0.96; Table 5).

**Table 4:** Respondents’ level of agreement that carbon program attributes impacted their willingness to join the carbon market and the number of respondents with any part of their land enrolled in a conservation easement

	Median response	Strongly disagree		Disagree		Neutral		Agree		Strongly agree	
		No.	%	No.	%	No.	%	No.	%	No.	%
The requirement to enroll in a conservation easement	Agree	48	10.96	43	9.82	123	28.08	117	26.71	107	24.43
The number of years required to be enrolled in the contract	Agree	41	9.38	27	6.18	60	13.73	142	32.49	167	38.22
The requirement to demonstrate soil carbon accumulation to earn credits	Neutral	41	9.60	33	7.73	193	45.20	118	27.63	42	9.84
A carbon program having an established track record of selling carbon credits	Neutral	35	8.27	24	5.67	195	46.10	112	26.48	57	13.48
The payment amount	Agree	33	7.76	19	4.47	96	22.59	129	30.35	148	34.82
The payment frequency	Agree	33	7.76	21	4.94	136	32.00	137	32.24	98	23.06
**Enrolled in a conservation easement**			**No**.					**%**			
Yes			94					19.38			
No			391					80.62

**Table 5:** Results from the chi-square test assessing if the four survey versions were equally distributed amongst respondents

	χ^2^	df	*p* value
Distribution of four survey versions	0.32	3	0.96

### Carbon Program Preferences

**Fig. 3:**
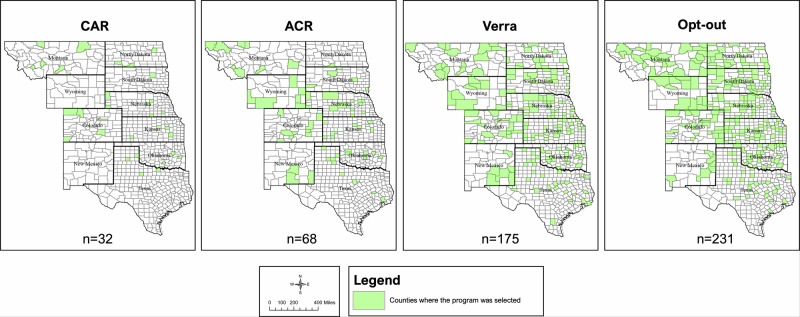
The counties and states in the Front Range and Great Plains, USA, where each carbon program was selected, and the number of counties in which each program was selected

Willingness to join varied substantially by the carbon program: 31% of the time respondents selected Verra’s carbon program (*n* = 583), compared to just 7% for ACR (*n* = 128) and 4% for CAR (*n* = 72; Table 6). However, most respondents chose to opt-out rather than enroll in any of the carbon programs (*n* = 1103; 58%). Verra had the broadest geographic reach, with ranchers in 175 different counties selecting it, compared to 68 for ACR and 32 for CAR (Fig. 3).

**Table 6:** The distribution of survey versions received, and the number of respondents who selected each carbon program or the opt-out

	No.	%
**Survey version** (*n* = 506)		
1	131	25.89
2	128	25.30
3	124	24.51
4	123	24.31
**Carbon program selection** (*n* = 1886)^a^		
CAR	72	3.82
ACR	128	6.79
Verra	583	30.91
Opt-out	1103	58.48

^a^Each survey had four choice experiments, thus four potential responses per respondent

Respondents’ agreement that specific carbon program attributes would influence their willingness to enroll significantly shaped their preferences for each carbon program. For CAR, ranchers who more strongly agreed that the number of years required in the contract impacted their willingness to participate had lower likelihood of selecting CAR compared to opting out (β = –0.35; *p* = 0.030; odds ratio = 0.70; Tables 7; 8). Interestingly, those who agreed that the requirement to demonstrate soil carbon accumulation would affect their willingness to enroll had higher likelihood of selecting CAR over opting out (β = 0.35; *p* = 0.046; odds ratio = 1.42). When all program attributes are held neutral, the likelihood of selecting CAR over opting out were 6%. Across all programs, respondents who had part of their land enrolled in a conservation easement were more likely to select a program compared to opting out.

**Table 7:** Results from the multinomial logistic regression models exploring the impact of the carbon program’s attributes (model 1), the payment frequency (model 2), and the payment amount (model 3) on respondents’ willingness to join a carbon program versus opting out

Predictor	Coefficient (β)	Standard error	z-value	*p* value	Odds ratio	95% CI^a^
**Model 1**: Impact of carbon program attributes x carbon program selected ^b,c^						
**CAR’s carbon program**						
Intercept	–2.78	0.17	–16.31	<0.001*	0.060	–3.11, –2.44
The requirement to enroll in a conservation easement	0.11	0.16	0.70	0.48	1.12	–0.20, 0.42
The number of years required to be enrolled in the contract	-0.35	0.16	–2.23	0.030*	0.70	–0.66, –0.043
The requirement to demonstrate soil carbon accumulation	0.35	0.17	2.00	0.046*	1.42	0.0066, 0.69
The established track record of selling carbon credits	–0.027	0.17	–0.13	0.90	0.98	–0.36, 0.31
Enrolled in a conservation easement	0.94	0.31	3.04	0.0023*	2.56	0.33, 1.55
**ACR’s carbon program**						
Intercept	–2.28	0.14	–16.82	<0.001*	0.10	–2.55, –2.02
The requirement to enroll in a conservation easement	-0.16	0.10	–1.57	0.12	0.85	–0.37, 0.040
The number of years required to be enrolled in the contract	-0.20	0.11	–1.83	0.068	0.82	–0.42, 0.015
The requirement to demonstrate soil carbon accumulation	0.086	0.12	0.70	0.48	1.09	–0.15, 0.33
The established track record of selling carbon credits	0.38	0.12	3.16	0.020*	1.47	0.14, 0.62
Enrolled in a conservation easement	1.25	0.22	5.67	<0.001*	3.47	0.81, 1.68
**Verra’s carbon program**						
Intercept	–0.66	0.074	-9.00	<0.001*	0.51	–0.81, –0.52
The requirement to enroll in a conservation easement	–0.12	0.060	–2.06	0.039*	0.88	–0.24, –0.0060
The number of years required to be enrolled in the contract	0.024	0.063	0.38	0.70	1.02	–0.10, 0.15
The requirement to demonstrate soil carbon accumulation	0.31	0.072	4.27	<0.001*	1.36	0.17, 0.45
The established track record of selling carbon credits	–0.027	0.072	–0.38	0.71	0.97	–0.17, 0.11
Enrolled in a conservation easement	0.63	0.14	4.50	<0.001*	1.88	0.36, 0.90
**Model 2:** Payment frequency ^d^ x carbon program selected						
**CAR’s carbon program**						
Intercept	–12.90	7.35	–1.75	0.080	0.28	–27.31, 1.52
Payment frequency	21.66	13.75	1.58	0.12	8.73	–5.28, 48.61
**ACR’s carbon program**						
Intercept	–12.96	7.35	–1.76	0.078	0.27	–27.38, 1.45
Payment frequency	21.91	13.75	1.59	0.11	8.95	–5.04, 48.86
**Verra’s carbon program**						
Intercept	–11.34	7.35	–1.54	0.12	0.32	–25.75, 3.07
Payment frequency	21.87	13.75	1.59	0.11	8.91	–5.07, 48.82
**Model 3:** Payment amount ^e^ x carbon program selected						
**CAR’s carbon program**						
Intercept	–10.59	2.15	–4.94	<0.001*	0.35	–14.80, –6.39
Payment amount	14.39	3.49	4.12	<0.001*	4.22	7.55, 21.23
**ACR’s carbon program**						
Intercept	–10.44	2.14	–4.87	<0.001*	0.35	–14.65, –6.24
Payment amount	14.43	3.49	4.13	<0.001*	4.23	7.59, 21.27
**Verra’s carbon program**						
Intercept	–8.85	2.14	–4.13	<0.001*	0.41	–13.04, –4.65
Payment amount	14.42	3.49	4.13	<0.001*	4.23	7.58, 21.27

^a^Wald 95% confidence intervals

^b^The dependent variable was the carbon program that the respondent selected (CAR, ACR, Verra, or opt-out). The opt-out was set as the reference level, thus every predictor shows the odds of a rancher selecting a carbon program compared to the odds of opting out

^c^The predictors in model 1 represent responses to the question, “To what extent do you agree that the following items would affect your willingness to enroll in a carbon market program?” and is coded as –2 = strongly disagree, –1 = disagree, 0 = neutral, 1 = agree, 2 = strongly agree

^d^Payment frequency varied between each choice experiment question, but could be annually, every 3 years, or every 5 years

^e^Payment amount varied between each choice experiment, but ranged from $1/acre to $30/acre. The odds ratios are reported per $0.10 increase in payment amount

^*^p < 0.05

**Table 8:** The multinomial logistic regression predictors’ effects on the log-odds (likelihood) of selecting each carbon program compared to opting out based on the results from the multinomial logistic regressions in Table 7

Predictor^†^	CAR’s carbon program	ACR’s carbon program	Verra’s carbon program
The requirement to enroll in a conservation easement	NS	NS	↓
The number of years required to be enrolled in the contract	↓	NS	NS
The requirement to demonstrate soil carbon accumulation	↑	NS	↑
The established track record of selling carbon credits	NS	↑	NS
Enrolled in a conservation easement	↑	↑	↑
Payment amount	↑	↑	↑
Payment frequency	NS	NS	NS

^†^ Responses to the question, “To what extent do you agree that the following items would affect your willingness to enroll in a carbon market program?” and is coded as –2 = strongly disagree, –1 = disagree, 0 = neutral, 1 = agree, 2 = strongly agree

↑ = increased log-odds (likelihood) of selecting the carbon program compared to opting out

↓ = decreased log-odds (likelihood) of selecting the carbon program compared to opting out

*NS* not significant

Respondents who agreed that an established track record of selling carbon credits would influence their willingness to participate had higher likelihood of selecting ACR over opting out (β = 0.38; *p* = 0.020; odds ratio = 1.47). When all program attributes are held neutral, the likelihood of a rancher selecting ACR over opting out were 10%.

For Verra, respondents who more strongly agreed that the requirement to demonstrate soil carbon accumulation would affect their willingness to participate had higher likelihood of selecting Verra’s carbon program compared to opting out (β = 0.31, *p* < 0.001; odds ratio = 1.36). Those who agreed that the conservation easement requirement would affect their willingness to participate were less likely to select Verra (β = –0.12; *p* = 0.039; odds ratio = 0.88). When all program attributes are held neutral, the likelihood of selecting Verra over opting out were 51%.

**Fig. 4:**
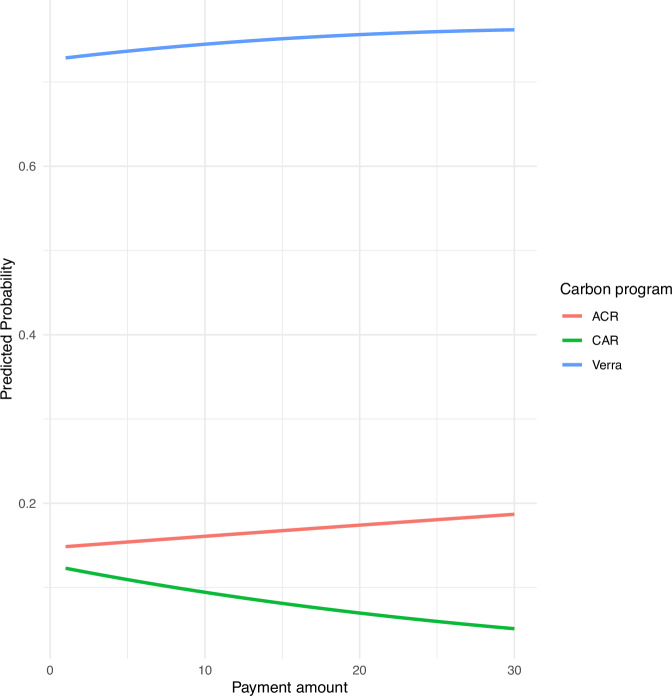
The predicted probabilities of ranchers selecting one of the three carbon programs (ACR, CAR, or Verra) at payment amounts ranging from $1/acre to $30/acre

Respondents who chose to participate in one of the three carbon programs reported an average required payment of $12.17 per acre (median = $6; standard deviation = $10.20), with contracts renegotiated every 2.87 years (median = 3 years; standard deviation = 1.91; Table 9). A $0.10 increase in payment increased the likelihood of a rancher selecting one of the carbon programs over opting out by more than four times. As payment amounts increase, the likelihood of a rancher selecting a carbon program under ACR or Verra also increases (Fig. 4). However, at the carbon credit prices presented here, the probability of rancher participation remains low for ACR and is especially low for CAR.

**Table 9:** Summary statistics for payment amount and payment frequency for each of the three carbon programs

	Median	Mean	Standard deviation	Minimum	Maximum	Skewness
Carbon program payment amount ($/acre)	6.00	12.17	10.20	1.00	30.00	0.76
Carbon program payment frequency (years)	3.00	2.87	1.91	1.00	5.00	0.13

Note: The table excludes those who opted out (they are coded as a 0 for both payment amount and payment frequency)

### Latent Class Analysis

A latent class analysis identified two distinct groups of respondents. Class 1, comprising 79% of the sample, was characterized by ranchers managing larger properties who primarily engaged in grazing and hay production, with a low likelihood of crop production and lower participation in conservation programs compared to class 2 (Table 10). In contrast, class 2 comprised 21% of respondents and represented smaller, more diversified operations that included crop and hay production, with all respondents having prior participation in conservation programs. Age and enrollment in a conservation easement did not show clear differentiation across classes, suggesting they did not explain any of the observed heterogeneity.

**Table 10:** The two latent class profiles and membership probabilities (%) based on respondent demographics and operation characteristics

Respondent characteristic	Class 1 (*n* = 536) membership probability (%)	Class 2 (*n* = 141) membership probability (%)
**Engages in crop production**		
Yes	9.57	100
No	90.43	0
**Engages in hay production**		
Yes	54.48	89.16
No	45.52	10.84
**Past participation in a conservation program**		
Yes	73.94	100
No	26.06	0
**Property size**		
Less than 2311 acres	36.69	65.55
Greater than 2311 acres	63.31	34.45
**Predicted class membership of sample**	79.17	20.83

Note: Chi-square goodness of fit = 36.58

**Fig. 5:**
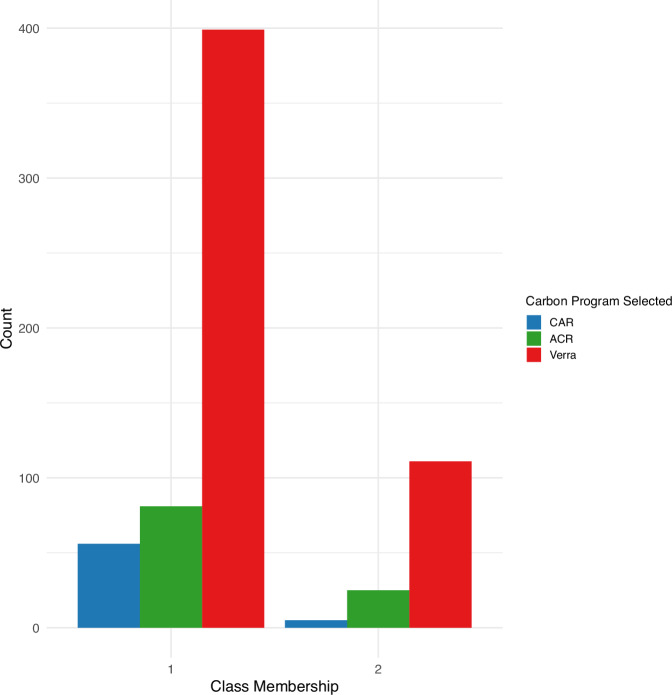
Respondents’ willingness to participate in each carbon program based on their assigned class membership determined by the latent class analysis. Ranchers in class 1 manage larger properties and primarily engage in grazing and hay production, while ranchers in class 2 operate smaller crop and hay operations and participate in conservation programs

The multinomial logistic regression revealed that class membership significantly influenced carbon program selection when CAR was the reference category (Table 11). Ranchers in class 2 were three times more likely to choose ACR over CAR compared to ranchers in class 1 (β = 1.10; *p* < 0.05), and 2.8 times more likely to choose Verra over CAR (β = 1.04; *p* < 0.05). Wilcoxon rank-sum tests revealed that ranchers belonging in class 2 were more likely to agree that the number of years required to enroll in the contract (w = 218,264; *p* < 0.001), a carbon program having an established track record of selling carbon credits (w = 218,504; *p* < 0.05), and the payment amount (w = 205,848; *p* < 0.001) influenced their willingness to join a carbon program (Table 12). Despite these differences, both classes shared the same ranking of carbon program preference: Verra was most preferred, followed by ACR, and then CAR (Fig. 5).

**Table 11:** The multinomial logistic regression results assessing how class membership and carbon program attributes impact carbon program preference

	Coefficient (β)	Standard error	z-value	*p* value	Odds ratio	95% CI^a^
**ACR’s carbon program**						
Intercept	–0.29	0.29	–1.03	0.31	0.75	–0.85, 0.27
Class membership	1.10	0.53	2.07	0.039*	3.00	0.06, 2.14
Affect_accumulate	–0.25	0.23	–1.09	0.28	0.78	–0.71, 0.20
Affect_contract	0.19	0.21	0.91	0.36	1.21	–0.22, 0.59
Affect_easement	–0.28	0.19	–1.47	0.14	0.75	–0.66, 0.10
Affect_record	0.44	0.23	1.86	0.063	1.55	–0.024, 0.90
Payment*frequency	0.012	0.0037	3.11	0.0012*	1.01	0.0043, 0.019
Enrolled in a conservation easement	0.37	0.39	0.95	0.34	1.45	–0.40, 1.14
**Verra’s carbon program**						
Intercept	1.44	0.23	6.27	<0.001*	4.21	0.99, 1.89
Class membership	1.04	0.49	2.12	0.034*	2.83	0.079, 2.00
Affect_accumulate	0.11	0.20	0.57	0.57	1.12	–0.28, 0.501
Affect_contract	0.45	0.18	2.49	0.013*	1.56	0.10, 0.80
Affect_easement	–0.33	0.17	–1.91	0.056	0.72	–0.66, 0.0088
Affect_record	–0.10	0.20	–0.49	0.63	0.91	–0.49, 0.29
Payment*frequency	0.011	0.0034	3.32	<0.001*	1.01	0.0046, 0.018
Enrolled in a conservation easement	-0.28	0.34	–0.81	0.42	0.76	–0.95, 0.39

Note: The dependent variable was the carbon program that the respondent selected (ACR, CAR, or Verra). CAR was set as the reference level, thus every predictor shows the odds of a rancher selecting ACR or Verra relative to the odds of selecting CAR

^a^Wald 95% confidence intervals

^*^p < 0.05

**Table 12:** Results from Wilcoxon rank-sum tests examining the differences between class membership and respondents’ level of agreement that carbon program attributes impacted their willingness to join the carbon market

	Class 1 median	Class 1 mean	Class 2 median	Class 2 mean	w	*p* value
The requirement to enroll in a conservation easement	Agree	0.45	Agree	0.42	248,536	0.91
The number of years required to be enrolled in the contract	Agree	0.80	Agree	1.04	218,264	<0.001*
The requirement to demonstrate soil carbon accumulation to earn credits	Neutral	0.19	Neutral	0.23	237,680	0.81
A carbon program having an established track record of selling carbon credits	Neutral	0.29	Neutral	0.40	218,504	0.047*
The payment amount	Agree	0.74	Agree	1.03	205,848	<0.001*
The payment frequency	Agree	0.56	Agree	0.65	228,976	0.21

^*^p < 0.05

The model also revealed that in general, respondents who more strongly agreed that contract length impacted their willingness to join the carbon market were more likely to select Verra compared to CAR (β = 0.45; *p* < 0.05; odds ratio = 1.56; Table 11). We also found that the interaction between payment amount and payment frequency was significant for both ACR and Verra, suggesting that the effect of payment amount differs depending on payment frequency. We found no significant relationship between respondents’ agreement that the requirement to demonstrate soil carbon accumulation, the requirement to enroll in a conservation easement, or an established track record impacted their willingness to join ACR or Verra compared to CAR.

## Discussion

Ranchers exhibited differing levels of interest across the carbon programs, with the majority choosing to opt-out rather than enroll (58%). Among those who did choose to participate, Verra’s carbon program was clearly the most preferred, followed by ACR and then CAR. These findings are contrary to actual registration, where CAR has the most number of projects registered. However, our results indicate that the remaining rancher population in the surveyed region not currently enrolled in a carbon program is unlikely to choose a program with long contract lengths and a requirement to enroll in a conservation easement. This reflects a common theme in working lands conservation: the importance of maintaining operational flexibility for future generations (Rissman et al. 2013; Stroman and Kreuter 2014). Our first model indicated that some respondents preferred carbon programs with a track record of carbon credit sales, likely because this signals a trustworthy reputation and a credible pathway to generate income that minimizes risk. Additionally, the preference for carbon programs that require measurable soil carbon accumulation suggests some ranchers value tangible, outcome-based verification of their land stewardship efforts. Those with some of their land enrolled in a conservation easement were more likely to select one of the carbon programs compared to those not enrolled in an easement. These findings aligns with literature suggesting landowners enrolled in conservation easements are more likely to engage in pro-environmental behavior and conservation programs (Farmer et al. 2011; Ma et al. 2012).

Payment amount significantly impacted enrollment in all three carbon programs. Although payment frequency alone did not significantly impact carbon program selection, payment amount appeared to be the most significant predictor of willingness to enroll in a carbon program. This suggests that time value preferences for receiving payment annually versus waiting to receive payment later as a lump sum was not a significant factor. This is counter to what one might expect given concepts related to time value of money and having more immediate cash flow. As payment amount increased, the probability of a rancher selecting a program also increased. Respondents who chose to enroll in one of the carbon programs accepted an average payment of $12.17 per acre, with contract renegotiation occurring every 2.87 years. The significant interaction between payment amount and payment frequency in the last model was positive, suggesting overall that more money is preferred to less, rather than capturing preference for payment frequency. These results together suggest that overall financial return is more important than cash flow timing. Given that we found relatively low enrollment rates, these results suggest that current carbon programs may not provide the adequate compensation necessary to motivate widespread rancher participation.

Respondents operating smaller, more diversified operations that included crop and hay production and with all respondents having prior participation in conservation programs were more likely to enroll in ACR or Verra. This preference may reflect the difficulty of committing cropland to a conservation easement with a 100-year contract, a central requirement of CAR. In contrast, those managing larger properties focused primarily on grazing and hay production, with no cropping and lower participation in conservation programs expressed a stronger relative preference for CAR. This may be because their land is already in perennial grass cover, making the carbon program’s 100-year contract less of a constraint. The conservation easement requirement may pose less of a barrier when long-term grassland use aligns with their existing management goals, reducing the perceived importance of contract length and payment amount. Others have also found a positive association between property size and engagement in longer term sustainable agricultural practices (Block, Danne, and Mußhoff 2024; Paulus et al. 2022).

One potential strategy to broaden the appeal of carbon programs is to stack them with existing government conservation programs, such as those in the U.S. (i.e., Grassland Conservation Reserve Program, Agricultural Management Assistance, Conservation Innovation Grant). Many ranchers are already enrolled in conservation programs (Nimlos et al. 2025a) and could simultaneously participate in a carbon program to supplement their income or cost-share ranch improvements. The programs listed above may also serve as alternatives for ranchers who opted out of carbon programs but remain interested in participating in federally administered conservation programs that tend to be less restrictive and more established than those in the carbon market. However, the availability of funding for these federal programs depends on Farm Bill reauthorizations and discretionary appropriations. In contrast, voluntary carbon markets are financed by private entities and may offer longer-term opportunities if trust and transparency can be improved.

ACR’s and CAR’s programs may align well with producers already interested in placing their land under a conservation easement. These carbon programs generate carbon credits by preventing the loss of soil carbon that could result from tillage, overgrazing, or land conversion. In such cases, landowners could benefit financially from the easement itself while also receiving payments for carbon credits. However, carbon programs requiring a conservation easement depend on operating in regions where there is high risk of conversion of grasslands to cropland (Nimlos et al. 2025b).

In 2024, agriculture was the only sector in the carbon market to experience an increase in carbon credit prices, with demand exceeding supply (Ecosystem Marketplace 2025). This trend suggests room for continued growth in agriculture-related carbon markets. The challenge for carbon companies is to find a balance between ensuring long-term soil carbon storage (permanence) and reducing barriers to participation, all while building landowner trust through greater transparency. Uncertainty surrounding contract terms, verification requirements, payment structures, and long-term obligations remains a significant barrier to carbon market enrollment. Increasing transparency regarding expected management changes, monitoring protocols, payment timelines, and potential risks could improve producers’ ability to evaluate whether participation in a carbon program is a good fit for their operation. Given that an established track record of selling carbon credits was related to increased enrollment in certain carbon programs, carbon companies should consider sharing case studies or success stories that clearly outline required management changes and payment structures.

There are several limitations to note. First, respondents had limited information about each program, potentially making it difficult to fully assess the relative advantages of one protocol over another. We described ACR and CAR as requiring conservation easements. Although the most common management change, conservation easements are not strictly required under these protocols. Due to space limitations in the survey, we did not provide respondents with detailed technical explanations of soil carbon quantification methods. Instead, we presented the attribute describing verification of soil organic carbon accrual at a high level to evaluate whether the requirement for soil sampling might influence landowners’ willingness to participate. As a result, respondents may not have fully understood the trade-offs between modeling versus direct soil sampling. Our study focused on three specific protocols, and these results may be different for other programs. Finally, although not assessed here, the potential for carbon market participation may be constrained by regional soil carbon sequestration capacity. For example, arid climates with limited rainfall may not support profitable levels of soil carbon accrual (Derner et al. 2019), potentially reducing landowner interest.

## Conclusion

As the number of carbon programs available to ranchers increases, understanding their preferences for program design is essential. Our survey of 506 ranchers managing more than 1.7 million acres revealed that most would prefer to opt-out of the carbon market rather than enroll. Those willing to participate preferred programs with shorter contract lengths and no conservation easement requirements. While some ranchers showed no strong preference between lump-sum and annual payments, payment amount significantly influenced their willingness to participate. Ranchers who also engaged in some crop production were more likely to prefer shorter programs, while larger grazing operators showed a relative preference for longer programs with a conservation easement requirement.

The most common barriers to enrollment were long contracts, low payment amounts, and the requirement of a conservation easement. Realistically, only ranchers already considering a conservation easement are likely to enroll in programs like CAR or ACR. Low overall willingness to participate likely stems from widespread skepticism toward carbon markets (Nimlos et al. 2025a), as well as concerns that program requirements may limit flexibility and impose burdensome restrictions.

To address these concerns, carbon companies should clearly communicate what management changes are required for enrollment (i.e., changes to grazing management or halting crop or hay production) and potential financial benefits. Negative perceptions of carbon markets likely overshadow success stories. Companies should also emphasize potential non-financial benefits, including land monitoring, improved land management plans, cost-share for grazing infrastructure, and marketing opportunities. Improving trust and transparency will be critical to increasing participation among ranchers.

## Data Availability

The data that support the findings of this study are not openly available due to reasons of sensitivity and confidentiality.
